# Supplementary material for: PCA-Driven Multivariate Trait Integration in Alfalfa Breeding: A Selection Model for High-Yield and Stable Progenies
Source: Plants (Basel). 2025 Sep 18;14(18):2906. doi: 10.3390/plants14182906 (PMC12473214; doi:10.3390/plants14182906)
Supplement: Supplementary file 1 [file plants-14-02906-s001.zip › Figure S1 Scree Plot of Principal Components for Six Agronomic Traits.pdf]

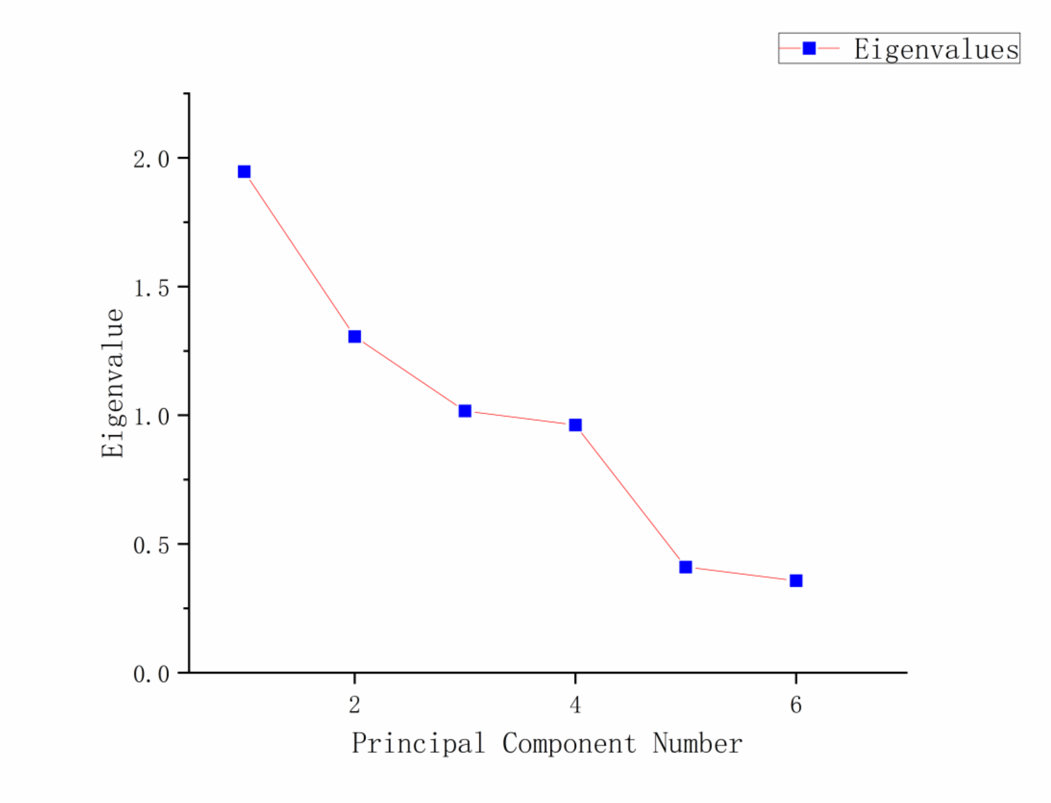

Figure S1 Scree Plot of Principal Components for Six Agronomic Traits. The scree plot displays the eigenvalues associated with each principal component extracted from six agronomic traits. According to the Kaiser criterion (eigenvalue > 1) and the elbow method, the first three principal components (PC1–PC3) were retained for further analysis.
